# Comparative Characterisation of Five Plasma-Derived Human Serum Albumin Preparations: Structural Integrity, Functional Properties, and Quality Attributes

**DOI:** 10.3390/ph19091470

**Published:** 2026-09-16

**Authors:** Zeineb Farhane, Melanie Bilong, Maud Le Boedec, Victor Blanchard, Arthur Besle, Nolwenn Tilly, Alexander Seifert

**Affiliations:** LFB Biotechnologies, 91940 Les Ulis, Francetillyn@lfb.fr (N.T.)

**Keywords:** human serum albumin, pharmaceutical quality, post-translational modifications

## Abstract

**Background:** Human serum albumin (HSA) is a critical therapeutic protein with widespread clinical applications. Despite standardised manufacturing protocols, quality variations between HSA preparations are poorly characterised. **Objective:** To comprehensively characterise five commercial plasma-derived albumin preparations (VIALEBEX^®^, ALBUNORM^®^, ALBUREX^®^, FLEXBUMIN^®^, ALBUTEIN^®^) using multiple analytical approaches to assess structural integrity, post-translational modifications, functional properties and purity. **Methods:** Ten batches in total from five manufacturers were analysed using size-exclusion chromatography (SEC) for aggregate quantification, ion exchange chromatography (IEX) for isoform profiling, surface plasmon resonance (SPR) for neonatal Fc receptor (FcRn) binding, and liquid chromatography-tandem mass spectrometry (LC-MS/MS) for protein quantification. **Results:** VIALEBEX^®^ contained the highest proportion of native HSA (35.2% vs. 15.1–22.2%) and the lowest levels of oxidised forms (13.2% vs. 27.4–33.3%) despite higher polymer content. SPR demonstrated higher FcRn binding affinity for VIALEBEX^®^. All preparations exceeded 98% albumin purity, with varying co-purified protein profiles. **Conclusions:** Significant quality variations exist between HSA preparations. VIALEBEX^®^ demonstrated greater structural integrity and functional properties despite a higher polymer content, suggesting complex quality relationships beyond simple aggregation. The preservation of structural integrity and higher redox state of VIALEBEX^®^ could be relevant considerations when selecting a product for clinical or pre-clinical use.

## 1. Introduction

Human serum albumin (HSA) is the most abundant plasma protein. It maintains critical physiological functions, including regulation of oncotic pressure, molecular transport, and antioxidant activity [1,2]. The single free cysteine at position 34 (Cys34), located on the albumin surface within domain I, is a primary plasma thiol reservoir and serves as the major extracellular antioxidant system [3,4,5]. In healthy individuals, approximately 70–80% of albumin has a reduced Cys34 (here called native or non-cysteinylated), while 20–30% exist in a reversibly oxidised form. Irreversible oxidation to sulphinic or sulphonic acid causes functional impairment [6].

Clinical applications of HSA are extensive, including volume replacement in hypovolaemic shock to restore oncotic pressure, for example during severe burns and surgical blood loss, management of ascites in liver disease, therapeutic plasma exchange, and as an excipient in pharmaceutical formulations [7,8]. In acute administration, HSA restores antioxidant activities in conditions like acute respiratory distress syndrome [5,8]. In long-term therapy HSA can be used to improve survival in decompensated cirrhosis [9,10,11]. Plasma-derived HSA has a clinical safety record spanning seven decades. There have been no documented transfusion-transmitted infections since the introduction of pasteurisation in the 1940s, confirming that it is a safe and reliable therapeutic agent [12].

Despite standardised manufacturing based on cold ethanol fractionation or some variation thereof, the quality of plasma-derived HSA products varies significantly between manufacturers [2,6,13]. Different fractionation steps may contribute to dimer and multimer formation, though ethanol-fractionated products typically maintain higher thiol-redox states [12]. Different fractionation protocols may also yield distinct proteoform profiles, potentially contributing to quality variations between products [14].

All analysed products have the same shelf-life of 3 years. However, during long-term storage, progressive chemical and redox modifications of HSA occur despite its overall structural stability. Quantitative analyses of commercial HSA preparations show substantially higher levels of thiol oxidation and carbonylation compared with albumin freshly isolated from healthy plasma, demonstrating that post-manufacture ageing contributes measurably to differences in quality [15].

Products well within the current pharmacopoeia standards can still show substantial differences in quality [16]. For instance, significant variations have been observed in free cysteine-34 content, which is directly correlated with radical-scavenging capacity [5]. The current study presents a comprehensive characterisation of five HSA products using orthogonal analytical techniques to assess structural integrity, post-translational modifications, functional properties and purity profiles.

## 2. Results

### 2.1. Size-Exclusion Chromatography Analysis

**Figure 1:**
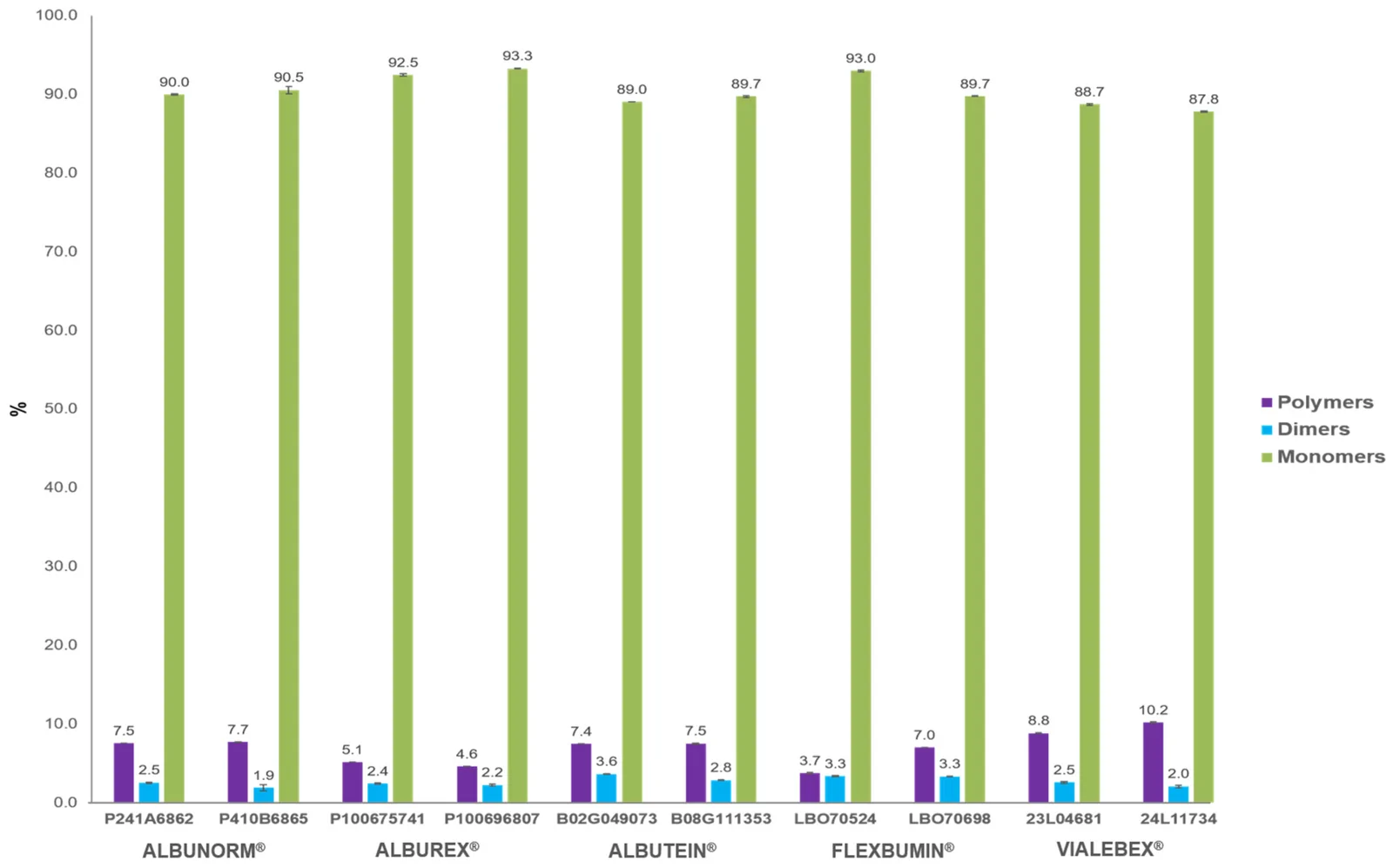
Percentages of polymers, dimers and monomers in albumin preparations.

All preparations showed the characteristic three-peak pattern corresponding to polymers, dimers, and monomers in order of elution. Size-exclusion chromatography (SEC) analysis revealed distinct aggregation profiles among the five albumin products (Figure 1). VIALEBEX^®^ demonstrated higher polymer content compared to other products, while maintaining substantial monomeric albumin levels (polymer content > 8% for VIALEBEX^®^ vs. 3.7–7.7% for comparators; monomers: VIALEBEX^®^ 87.8–88.7% vs. 89.0–93.3% for comparators).

### 2.2. Ion Exchange Chromatography Isoform Profiling

**Figure 2:**
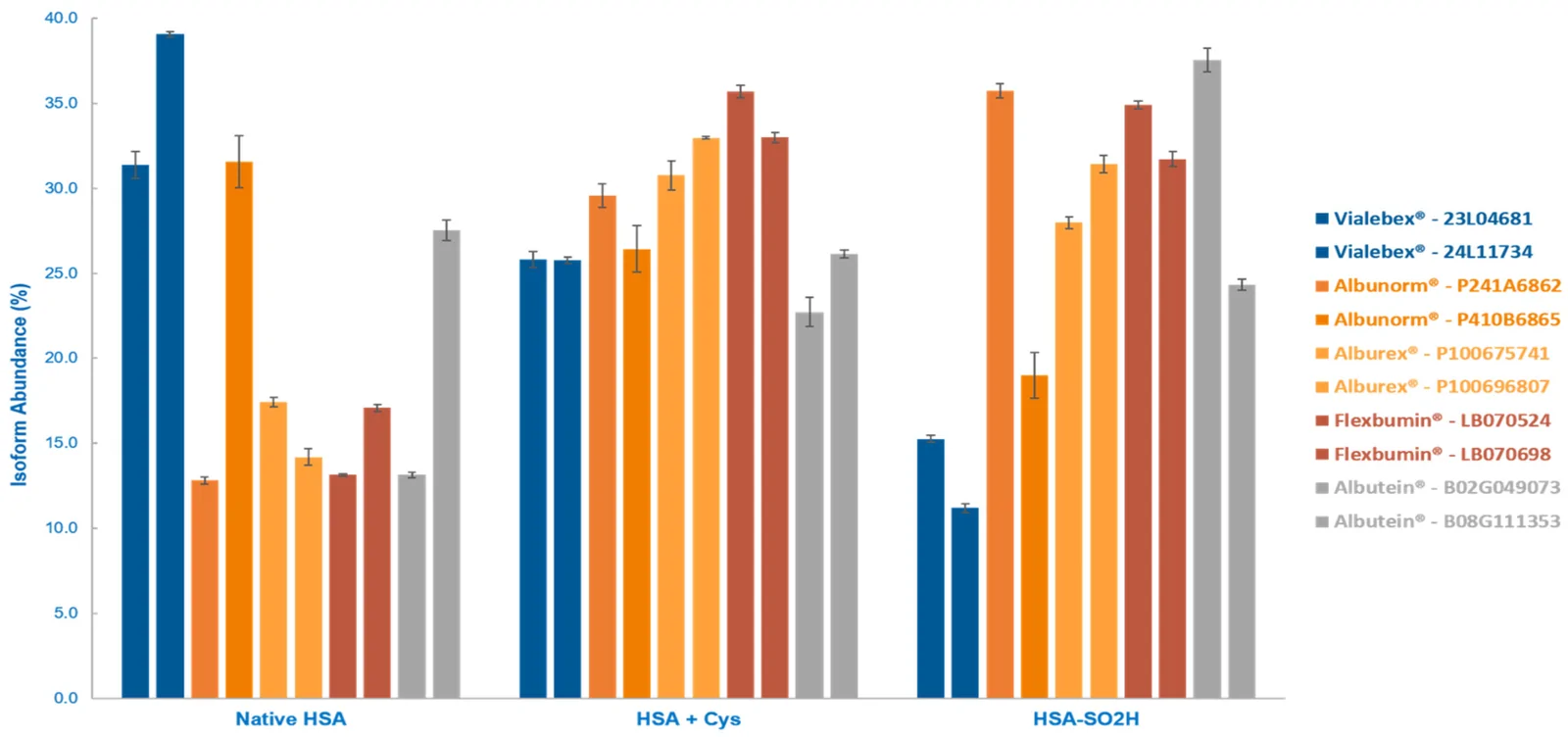
Isoform abundance by ion-exchange chromatography demonstrating isoform heterogeneity across products.

Ion-exchange chromatography (IEX) analysis revealed substantial heterogeneity in post-translational modification patterns across products (Table 1, Figure 2). VIALEBEX^®^ contained the highest proportion of native HSA (35.2 ± 5.4% vs. 15.1 ± 2.2) and lowest levels of oxidised forms (13.2 ± 2.9% vs. 33.3 ± 1.8%). FLEXBUMIN^®^ exhibited the lowest native form percentage and the highest oxidised form percentage. VIALEBEX^®^ had slightly more truncated forms than comparators. ALBUTEIN^®^ and ALBUNORM^®^ showed higher batch-to-batch variability. All analysed products showed comparable amounts of cysteinylated forms.

### 2.3. Comparison with Human Plasma Reference

**Figure 3:**
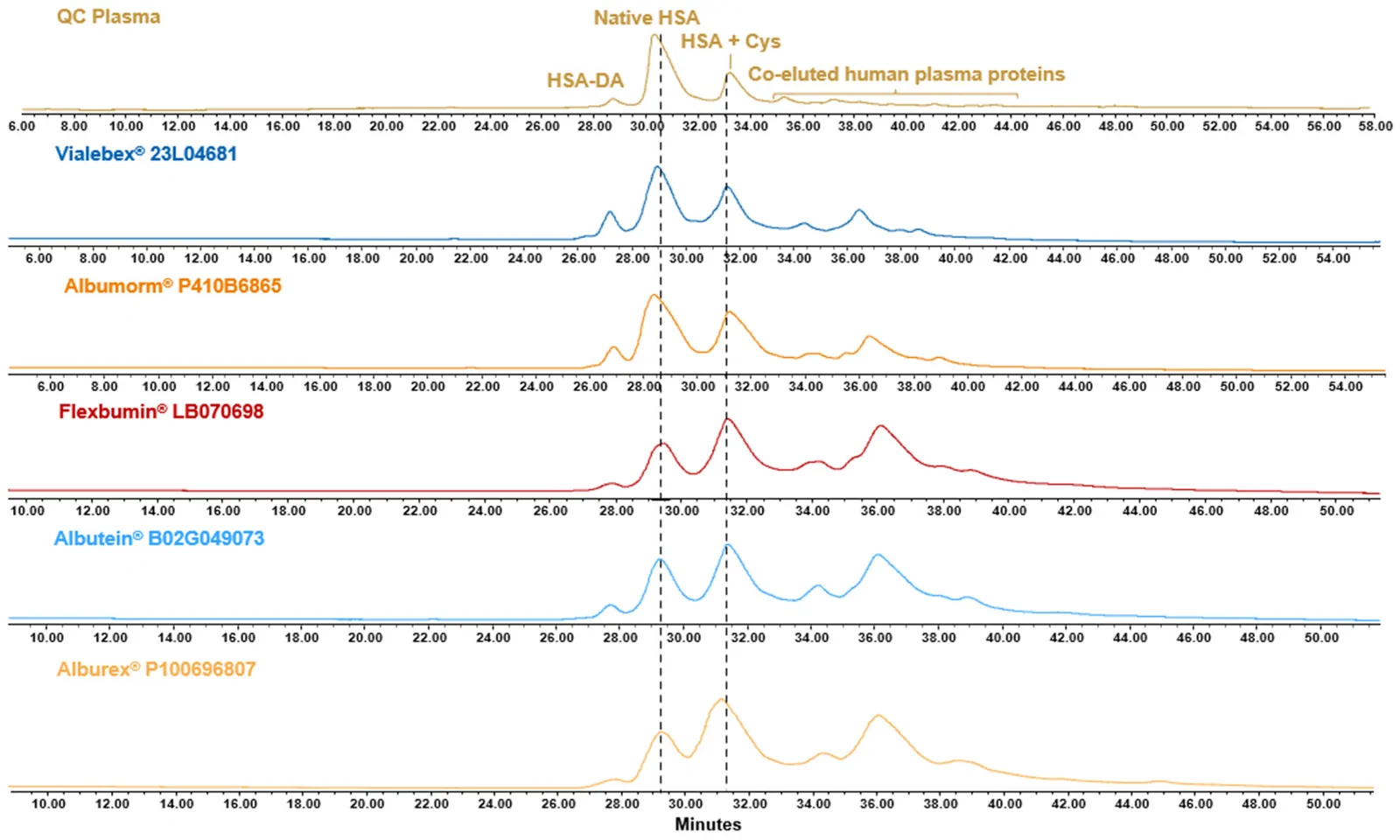
Albumin isoform distribution on IEX: therapeutic products vs. plasma reference. Cys: cysteine; DA: truncated form; HSA: human serum albumin; IEX: ion-exchange chromatography.

Comparison with quality control plasma revealed all therapeutic preparations contained lower native HSA and higher oxidised forms than fresh plasma (51.3% vs. 15.8–35.7%) (Table 2, Figure 3). The presence of irreversibly oxidised HSA-SO_2_H in all HSA preparations, which was not detected in fresh plasma where the oxidised form may co-elute with small amounts of other human plasma proteins, indicates cumulative oxidative stress during processing and storage. VIALEBEX^®^ was the closest to albumin in human plasma in terms of quantities of native and cysteinylated isoforms.

### 2.4. Surface Plasmon Resonance: FcRn Receptor Binding

**Figure 4:**
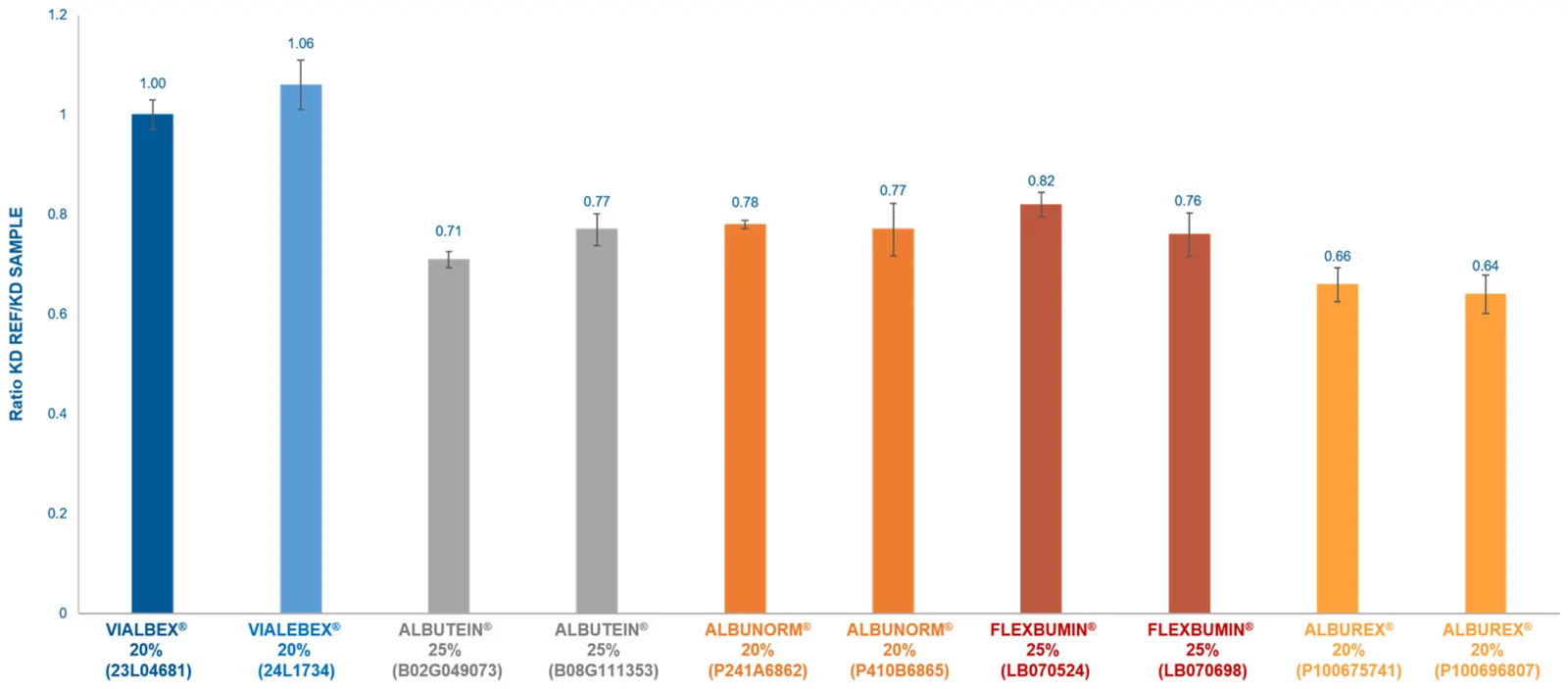
Surface plasmon resonance of normalised KD Ratios of albumin-FcRn interaction at pH 6.0. FcRn: neonatal Fc receptor.

Surface plasmon resonance (SPR) analysis demonstrated differential binding kinetics to the neonatal Fc receptor (FcRn), which mediates albumin recycling and determines plasma half-life (Figure 4). It should be noted that albumin half-life is modulated by multiple factors in addition to FcRn-mediated recycling. VIALEBEX^®^ has a higher affinity towards the FcRn receptor than comparators (results normalised for VIALEBEX^®^ batch 23L04681: 1.00 vs. 0.64–0.83). This difference in affinity was mostly driven by a higher association rate. The different sensograms are shown in the Supplementary Materials, Figure S1.

The FcRn–albumin interaction is pH-dependent and critical for maintaining albumin’s long circulation time of approximately 19 days [4]. VIALEBEX^®^ exhibited greater binding affinity compared to other HSA products (KD: 3.61 vs. 4.98–5.79 µM), suggesting potential for enhanced in vivo stability and longer circulation time (Table 3).

### 2.5. LC-MS/MS Relative Protein Quantification

Using liquid chromatography–tandem mass spectrometry (LC-MS/MS) top-three peptide relative quantification (Hi3), albumin represented more than 98% of the detectable protein content in all samples, consistent with a high degree of purity (Supplementary Materials, Table S1).

### 2.6. Co-Purified Proteins

**Figure 5:**
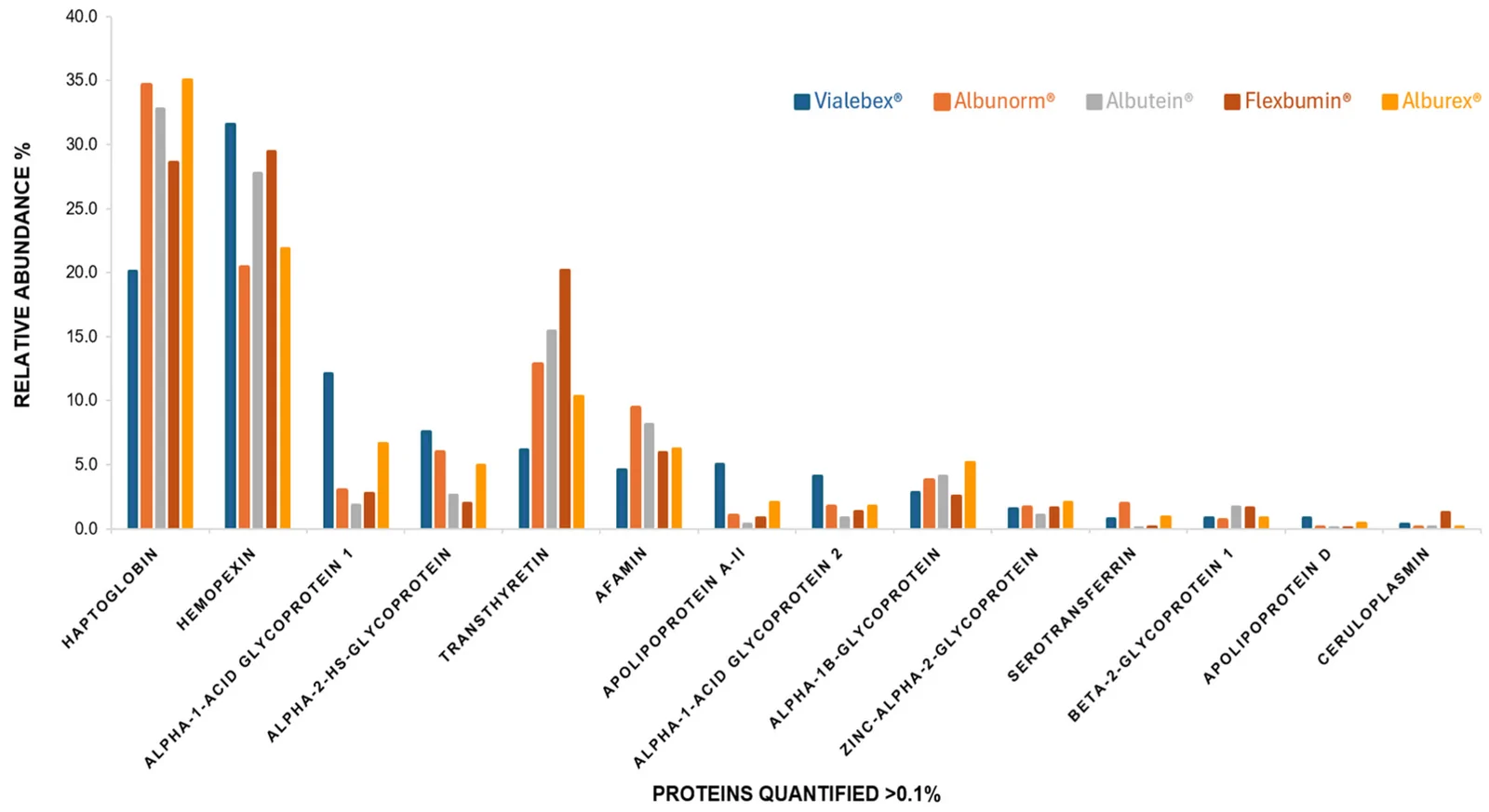
Relative abundance of co-purified non-albumin proteins.

Analysis of accompanying proteins revealed distinct impurity profiles (Figure 5).

The same co-purified proteins were found in all albumin products but in different proportions which could be related to some difference in the purification processes.

VIALEBEX^®^ had less haptoglobin than the other products, but higher hemopexin, alpha glycoprotein 1 and 2. FLEXBUMIN^®^ had higher transthyretin and ceruloplasmin levels.

## 3. Discussion

This comprehensive analytical characterisation reveals substantial variations in structural and functional integrity among HSA preparations. The observed higher preservation of albumin structural and conformational integrity in VIALEBEX^®^, despite it having the highest number of polymers, suggests that HSA quality includes multiple interdependent attributes that collectively may affect therapeutic efficacy.

Higher FcRn binding affinity directly impacts pharmacokinetics through pH-dependent recycling, which protects albumin from lysosomal degradation [17,18]. The correlation between the observed higher native albumin content and enhanced FcRn binding in VIALEBEX^®^ suggests that structural integrity influences receptor interaction and plasma half-life, potentially reducing dosing frequency and improving therapeutic efficiency.

The preservation of native albumin (35.2% in VIALEBEX^®^ vs. 15.1–22.2% in other HSA products) is the most critical finding as this directly correlates with preserved redox capacity and structural integrity [19]. The free sulphinic group at Cys34 serves as the primary extracellular antioxidant, making its preservation essential for therapeutic efficacy in some clinical settings [5]. Structural studies have shown that Cys34 in isolated domain I achieves ~24-fold higher reactivity than in full-length protein, indicating that conformational constraints and post-translational modifications substantially reduce functional capacity [20]. Moreover, the degree of oxidised Cys34 in HSA is correlated with oxidative stress-related pathological conditions [21]. The level of oxidised albumin may increase up to 70% under pathologic conditions, like kidney or liver diseases [22].

The higher polymer content in VIALEBEX^®^ should be interpreted within a functional context. For example, not all aggregates are equally immunogenic or functionally compromised; smaller aggregates may be taken up and degraded by different pathways than larger aggregates. Smaller aggregates also tend to be more immunogenic than larger formations [23]. The pharmaceutical industry generally aims to minimise aggregate content, but functional assays provide more relevant quality assessment than aggregate percentage alone, particularly for applications requiring large volumes such as plasma exchange and management of hypovolaemic shock [13].

Plantier et al. [5] previously reported substantial inter-product variation in HSA antioxidant capacity; our IEX data extend those findings by linking that variation to specific isoform profiles. The >2-fold difference in native albumin content translates directly to enhanced redox capacity and may have significant effects on therapeutic efficacy. This is particularly critical in oxidative stress conditions like sepsis, acute respiratory distress syndrome, and ischaemia–reperfusion injury [5].

The elevated levels of irreversibly oxidised albumin observed in other HSA products suggest a loss of redox-active thiol functionality, which may reduce the antioxidant buffering capacity of plasma albumin and thereby exacerbate oxidative stress rather than actively promote redox cycling [6]. The correlation between higher oxidised albumin content and reduced FcRn binding affinity demonstrates that structural integrity may influence multiple functional parameters simultaneously, potentially improving pharmacokinetics through enhanced receptor recycling and extended plasma half-life [4]. This may have direct implications for dosing frequency and therapeutic efficiency in clinical applications, specifically for short-term supplementation in the prevention of circulatory dysfunction for example.

The consistency of albumin isoform profiles within products given the significant differences between manufacturers is highly suggestive of systematic variations in structural preservation rather than random batch-to-batch differences [24,25]. Manufacturing parameters including purification/biosafety conditions and stabiliser formulations can introduce modifications with distinct functional consequences: N-terminal truncation impairs metal chelating capacity, Cys34 oxidation eliminates antioxidant function, and glycation affects protein stability [3,4]. VIALEBEX^®^ is the only product formulated without N-acetyltryptophan and not a quantified amount of sodium caprylate; both known to be heat stabilisers, to prevent protein aggregation during heat treatment, and oxidative degradation preservatives. This likely explains the higher polymer content observed in VIALEBEX^®^, while the absence of these exogenous protectants suggests that the lower oxidation levels reflect intrinsic process-related preservation rather than excipient-mediated protection [26,27]. The “effective albumin concentration theory” suggests that only structurally intact albumin contributes therapeutically; thus, preparations that have undergone extensive processing may deliver substantially less bioactive albumin than the labelled concentration indicates [14,28,29]. These quality variations may have direct clinical relevance. Products with higher structural integrity and preserved redox state may provide enhanced therapeutic benefit in conditions characterised by oxidative stress and compromised antioxidant defences. The >2-fold variation in redox capacity represents a clinically meaningful difference that could potentially impact patient outcomes, particularly in critically ill patients where albumin serves both volume expansion and antioxidant protection roles.

Recently, long-term HSA therapy has been shown to be effective in decompensated cirrhosis, significantly improving survival in targeted patients [9,10,11]. Given this increasingly established and recommended use of HSA in long-term therapies, preserved structural integrity becomes essential to avoid cumulative oxidative burden from repeated infusions [7,30]. In addition, post-translational modifications alter drug binding sites, potentially affecting pharmacokinetics of co-administered medications [4].

Producing high purity HSA that does not contain additional proteins would require a purification process incompatible with the demand for therapeutic albumin. Accordingly, HSA products may contain up to 5% non-albumin proteins (95% purity is recommended by European pharmacopoeia) [24]. While proteomic analyses have identified numerous accompanying proteins in plasma-derived preparations, their significance must be evaluated within the broader context of structural and functional integrity [24,31]. The differences in relative abundance of co-purified protein among the albumin products may suggest differences in the purification processes of different manufacturers and may correlate with adverse effects in patients [25]. Although recent evidence suggests some plasma-derived accompanying proteins may confer beneficial properties absent in recombinant alternatives, the primary therapeutic advantage lies in albumin’s preserved structural and functional integrity [31]. Haptoglobin was the most abundant accompanying protein across all manufacturers, consistent with previous proteomic surveys [24,31]. VIALEBEX^®^ was found to have the lowest haptoglobin burden, in keeping with more thorough purification, while the remaining co-purified species, including afamin and other members of the albumin family [32], reflect normal plasma constituency rather than contamination, and several contribute complementary transport or antioxidant functions in vivo. Some accompanying proteins may modulate the disposition of co-administered drugs. Haemopexin, for example, can interact with arginine-rich therapeutic peptides beyond its canonical haem-scavenging role [33], which could be a clinical consideration in patients receiving concurrent polypharmacy and albumin infusion.

Current pharmacopeial standards inadequately address structural and functional integrity, focusing instead on basic purity parameters [8]. Advanced analytical techniques now enable assessment of functionally relevant attributes, including redox state and conformational stability [6,16,34]. The substantial differences revealed here demonstrate the need for expanded quality requirements that prioritise functional integrity over traditional purity metrics.

The major limitations of this study include the limited number of batches analysed, which may not capture the full manufacturing variability across production cycles (Supplementary Materials, Table S2). Another caveat is that in vitro measurements may not fully predict in vivo performance, and the absence of clinical data linking analytical parameters to patient outcomes prevents definitive therapeutic conclusions.

## 4. Materials and Methods

Ten HSA products batches were analysed: 2 batches of VIALEBEX^®^ 20% (LFB S.A./LFB Biomédicaments, Les Ulis, France); and 2 batches each of ALBUNORM^®^ 20% (Octapharma AG, Lachen, Schwyz, Switzerland), ALBUREX^®^ 20% (CSL Behring L.L.C, King of Prussia, PA, USA), FLEXBUMIN^®^ 25% (Baxalta US Inc., Bannockburn, IL, USA), and ALBUTEIN^®^ 25% (Grifols S.A., Sant Cugat del Vallès, Barcelona, Spain). Differences in HSA formulations with respect to stabilisers and sodium content are shown in the Supplementary Materials, Table S3.

An extended dataset of five VIALEBEX^®^ batches was also analysed to characterise production consistency and batch-to-batch variability; these results are reported in Supplementary Table S4.

All analytical measurements were performed in triplicate for each batch to ensure reproducibility and statistical validity. All albumin preparations were maintained under controlled and similar storage conditions (4 °C, protected from light) until analysis to minimise storage-related variability. The study was not performed blinded to product identities.

All analytical instruments were qualified and annually maintained by each respective manufacturer.

### 4.1. Size-Exclusion High-Performance Liquid Chromatography

Analysis was performed using a UltiMate™ 3000 high-performance liquid chromatography (HPLC) system (Thermo Fisher Scientific, Waltham, MA, USA) equipped with a TOSOH TSK G3000SWXL, 5 μm particle size, 7.8 × 300 mm column (TOSOH, Tokyo, Japan). Phosphate buffer was used for the mobile phase at a flow rate of 0.5 mL/min at room temperature and detection made in ultraviolet (UV) absorbance at 280 nm. Chromatographic peaks were integrated to determine relative percentages of polymers (high molecular weight aggregates), dimers, and monomers based on retention time and peak area. Polymers eluted first, followed by dimers, then monomeric albumin as the major peak.

### 4.2. Ion-Exchange Chromatography

IEX enables the separation of different albumin isoforms. This technique complements other methods such as chromatofocusing, which has been used to separate redox states [6]. IEX is the method of choice for characterising protein variants and specifically for analysing HSA isoforms. It has demonstrated ability to separate and quantify cysteinylated from non-cysteinylated forms and other isoforms, such as oxidised, glycated and deamidated [3]. This enables the assessment of structural integrity as a critical quality attribute in HSA preparations [3].

Isoform separation was achieved using an Acquity H-Class Bio UHPLC (Waters, Milford, MA, USA) with a Proteomix SAX-NP5, 2.1 × 150 mm, 5 μm, 300 Å column (Sepax Technologies, Newark, MA, USA). The mobile phase A binding buffer was 50 mM ammonium acetate; the mobile phase B elution buffer was 500 mM ammonium acetate. Flow rate was 0.2 mL/min at 30 °C and UV detection at 280 nm. The following isoforms were identified based on retention time and previous characterisation studies using mass spectrometry: HSA-DA: N-terminally truncated form lacking Asp-Ala dipeptide; native HSA: unmodified albumin with free Cys34; HSA + Hex: glycated form; HSA + Cys: cysteinylated albumin; HSA + Cys + Hex: cysteinylated and glycated form; HSA-DA-SO_2_H: truncated and sulphinic acid oxidised form; HSA + Cys-Gly: cysteinyl glycine adduct; HSA-SO_2_H: sulphinic acid oxidised form; and as an example, the mass spectrometry identification of the three major isoforms of one VIALEBEX^®^ batch is shown in Supplementary Figure S2.

### 4.3. Surface Plasmon Resonance Analysis

SPR was used to determine FcRn binding affinity. SPR experiments were performed using a Biacore T200 system (Cytiva, formerly GE Healthcare, Malborough, MA, USA) with a CAP chip with Biotin CAPture Kit (Cytiva, Malborough, MA, USA). The ligand was biotinylated human FcRn (ACROBiosystems, Newark, DE, USA) immobilised at ~150 RU via biotin-streptavidin capture with a running buffer of 67 mM sodium phosphate, 150 mM NaCl, 0.005% Tween 20, pH 6.0 at 25 °C. Serial dilutions of HSA (312.5 nM to 80 μM) were injected in single-cycle kinetic mode. Kinetic parameters (ka, kd, KD) were determined using Biacore evaluation software 3.2.2. Non-specific binding was corrected using reference flow cell subtraction and blank buffer injections. Each sample was analysed in triplicate, with reference samples in quadruplicate. Results were normalised relative to VIALEBEX^®^ batch 23L04681.

### 4.4. Liquid Chromatography-Tandem Mass Spectrometry

The sample preparation was performed at low pH with the AccuMAP™ Low pH Protein Digestion Kit from Promega Corporation (Madison, WI, USA). After denaturation, samples were reduced by TCEP (tris(2-carboxyethyl)phosphine), alkylated by IAA (iodoacetamide), pre-digested by Lys-C and digested by Lys-C and Trypsin. Peptides were eluted on a Vanquish Neo, Thermo Scientific instrument (Waltham, MA, USA) concentrated on a trap column (PEPMAP NEO C18, 5 µm, 300 µm × 5 mm) then separated on a C18 column (EASY-SPRAY PEPMAP NEO, 2 µm, 75 µm × 150 mm) before analysis by ESI+/DDA mass spectrometry on an Orbitrap Exploris 480 Thermo Scientific instrument (Waltham, MA, USA), Thermo Scientific Xcalibur v 4.7. Each sample was run in triplicate and results were averaged. The acquisition parameters were 275 °C for capillary transfer, 1900 V for ionisation, resolution: 60,000, scan range: 375–1500 *m*/*z*, dynamic exclusion 45 s, RF lens 50%, charge state: 2–5 and DDA 10 scans.

Peaks studio 11.0 Bioinformatics Solutions Inc. software search was performed to identify proteins with the following search parameters: Database: UniprotKB/Swissprot Homo sapiens; Enzyme: Trypsin; Missed cleavage allowed: 2; Parent mass error tolerance: 10 ppm; Fragment mass error tolerance: 0.5 Da; Charge: between 2 and 6; Fixed modification: cysteine carbamidomethylation; False Discovery Rate: less than 0.1% for peptides and 1% for proteins.

Progenesis QI for proteomics Nonlinear Dynamics 4.2 and Excel Microsoft software were used to calculate the relative abundance by Hi-3 analysis. After loading raw files in Progenesis QI for proteomics Bioinformatics Solutions, three parameters: intensity, retention time (alignment process score) and area (normalisation) were considered to assess the quality of the different runs. Only proteins identified with at least three peptides were considered.

### 4.5. Statistical Analysis

Results are presented as mean ± standard deviation. Each product was analysed using two batches (n = 2). All analyses were performed in triplicate (r = 3).

## 5. Conclusions

This multi-parametric characterisation demonstrates significant heterogeneity among HSA preparations in terms of structural integrity, post-translational modifications, and functional properties, despite meeting current purity standards. VIALEBEX^®^ demonstrated greater preservation of structural and conformational integrity, with the highest proportion of native albumin (35.2% vs. 15.1–22.2% in other HSA products), lowest irreversible oxidation (13.2% vs. 27.4–33.3% in other HSA products), and enhanced FcRn receptor binding affinity. The preservation of structural and conformational integrity, in addition to higher redox properties should be considered when selecting a product for the management of critical patients, where both volume support and antioxidant protection are essential for clinical and pre-clinical trials. These findings support expanding quality standards beyond simple purity metrics, to include functional assessments and redox state characterisation, ultimately improving the therapeutic efficacy of this essential plasma protein.

## Figures and Tables

**Table 1:** Relative quantification of albumin isoforms by ion exchange chromatography (%) *.

Isoform	VIALEBEX^®^	ALBUNORM^®^	ALBUREX^®^	FLEXBUMIN^®^	ALBUTEIN^®^
HSA-DA	7 ± 0.4	3 ± 2.6	2 ± 0.7	1.4 ± 0.4	3.1 ± 1.8
Native HSA	35.2 ± 5.4	22.2 ± 13.3	15.8 ± 2.3	15.1 ± 2.2	20.3 ± 10.2
HSA + Hex	3.4 ± 0	1.9 ± 0.5	1.8 ± 0.1	Not detected	1.9 ± 0.5
HSA + Cys	25.8 ± 0	28 ± 2.2	31.9 ± 1.6	34.3 ± 1.5	24.4 ± 2.4
HSA + Cys + Hex	6.9 ± 1	6.3 ± 0.6	7 ± 0.2	Not detected	6.6 ± 1.2
HSA-DA-SO_2_H	6.2 ± 1.4	8.2 ± 1.9	9 ± 0.7	10.8 ± 0.2	9.8 ± 3.6
HSA + Cys-Gly	2.2 ± 0.2	3 ± 0.1	2.8 ± 0.2	5 ± 0.7	2.8 ± 0.7
HSA-SO_2_H	13.2 ± 2.9	27.4 ± 11.8	29.7 ± 2.4	33.3 ± 1.8	30.9 ± 9.3

* Values represent mean ± SD. Cys: cysteinylated; Cys-Gly: cysteinylated and glycinylated; DA: truncated form; DA-SO_2_H: truncated and oxidised; HSA: human serum albumin; Hex: glycated; SD: standard deviation; SO_2_H: oxidised.

**Table 2:** Albumin isoform distribution: therapeutic products vs. plasma reference (%).

Sample	Native HSA	HSA + Cys	HSA-SO_2_H
QC plasma	51.3	25.5	Not detected
VIALEBEX^®^	35.2	25.8	13.2
ALBUNORM^®^	22.2	28.0	27.4
FLEXBUMIN^®^	15.1	34.3	33.3
ALBUTEIN^®^	20.3	24.4	30.9
ALBUREX^®^	15.8	31.9	29.7

Cys: cysteine; HSA, human serum albumin, SO_2_H: oxidised; QC: quality control.

**Table 3:** Binding affinities (KD, µM).

Product	KD (µM)
VIALEBEX^®^	3.61 ± 0.338
ALBUTEIN^®^	5.22 ± 0.098
ALBUNORM^®^	4.98 ± 0.190
ALBUREX^®^	5.53 ± 1.477
FLEXBUMIN^®^	5.79 ± 0.641

KD: equilibrium dissociation constant.

## Data Availability

The original contributions presented in this study are included in the article. Further inquiries can be directed to the corresponding author.

## Supplementary Materials

The following supporting information can be downloaded at https://www.mdpi.com/article/10.3390/ph19091470/s1, Figure S1: Surface plasmon resonance sensorgrams and residuals of albumin-FcRn interaction at pH 6.0; Figure S2: MS spectra of native, cysteinylated and oxidised albumin isoforms of VIALEBEX^®^. Inserts represent deconvoluted mass spectra; Table S1: Relative abundance of albumin proteins in HSA samples. Table S2: Analysed product batches; Table S3: Excipient composition and sodium content of human serum albumin preparations; Table S4: Five VIALEBEX^®^ batch analysis (%).

## Abbreviations

The following abbreviations are used in this manuscript:

Asp-Ala	aspartic acid–alanine dipeptide
C18	octadecyl-bonded silica
Cys	cysteine
Cys-Gly	cysteinyl-glycine
DDA	data-dependent acquisition
DA	truncated form
ESI	electrospray ionisation
FcRn	neonatal Fc receptor
Hex	hexose adduct (glycation)
Hi3	top-three peptide relative quantification
HPLC	high-performance liquid chromatography
HSA	human serum albumin
IAA	iodoacetamide
IEX	ion-exchange chromatography
KD	equilibrium dissociation constant
ka	association rate constant
kd	dissociation rate constant
LC-MS/MS	liquid chromatography–tandem mass spectrometry
Lys-C	lysyl endopeptidase C
NaCl	sodium chloride
QC	quality control
RU	response units
SD	standard deviation
SEC	size-exclusion chromatography
SPR	surface plasmon resonance
SO_2_H	sulphinic-acid-oxidised
TCEP	tris(2-carboxyethyl)phosphine
UHPLC	ultra-high-performance liquid chromatography
UV	ultraviolet

## References

[1] Beeram S.R., Zhang C., Suh K., Clarke W.A., Hage D.S. (2021). Characterization of drug binding with alpha(1)-acid glycoprotein in clinical samples using ultrafast affinity extraction. J. Chromatogr. A.

[2] Quinlan G.J., Martin G.S., Evans T.W. (2005). Albumin: Biochemical properties and therapeutic potential. Hepatology.

[3] Leblanc Y., Bihoreau N., Chevreux G. (2018). Characterization of Human Serum Albumin isoforms by ion exchange chromatography coupled on-line to native mass spectrometry. J. Chromatogr. B.

[4] Leblanc Y., Berger M., Seifert A., Bihoreau N., Chevreux G. (2019). Human serum albumin presents isoform variants with altered neonatal Fc receptor interactions. Protein Sci..

[5] Plantier J.L., Duretz V., Devos V., Urbain R., Jorieux S. (2016). Comparison of antioxidant properties of different therapeutic albumin preparations. Biologicals.

[6] Turell L., Botti H., Bonilla L., Torres M.J., Schopfer F., Freeman B.A., Armas L., Ricciardi A., Alvarez B., Radi R. (2014). HPLC separation of human serum albumin isoforms based on their isoelectric points. J. Chromatogr. B.

[7] Liu Q., Lin Z., Li C., Wang Z., Xu J., Cheng L., Ma L., Wang Z. (2024). Comparative Analysis of Post-Translational Modifications of Human Serum Albumin Derived from Human Plasma and Recombinant Sources in China. Discov. Med..

[8] Simon T., Schumann P., Bieri M., Schirner K., Widmer E. (2023). Hyperoncotic human albumin solutions for intravenous fluid therapy: Effectiveness of pathogen safety and purification methods, and clinical safety. Biosaf. Health.

[9] Caraceni P., Riggio O., Angeli P., Alessandria C., Neri S., Foschi F.G., Levantesi F., Airoldi A., Boccia S., Svegliati-Baroni G. (2018). Long-term albumin administration in decompensated cirrhosis (ANSWER): An open-label randomised trial. Lancet.

[10] Torp N. (2025). Evolving Paradigms in Albumin Therapy for Decompensated Cirrhosis: Highlights from the EASL Congress 2025. EMJ Hepatol..

[11] Fortea J.I., Rodríguez D.R., Albillos A., Vergés E.S., Bañares R. (2025). Clinical Perspectives on Long-Term Albumin Therapy in Decompensated Cirrhosis: A Nationwide Delphi Survey in Spain-The ALBA Study. Liver Int..

[12] Kelley W., Garcia E., Erdlenbruch W., Turecek P.L., Kreil T.R., Unger U., Hammad T.A., Petermann R. (2025). Donors to patients—A narrative review of safety and manufacturing of human serum albumin. Anal. Blood.

[13] Nakae H., Tomida K., Kikuya Y., Okuyama M., Igarashi T. (2017). Comparison of quality of human serum albumin preparations in two pharmaceutical products. Acute Med. Surg..

[14] Woodland B., Coorssen J.R., Padula M.P. (2024). Protein “purity,” proteoforms, and the albuminome: Critical observations on proteome and systems complexity. Front. Cell Dev. Biol..

[15] Takahashi T., Terada T., Arikawa H., Kizaki K., Terawaki H., Imai H., Itoh Y., Era S. (2016). Quantitation of Oxidative Modifications of Commercial Human Albumin for Clinical Use. Biol. Pharm. Bull..

[16] Maeyama L., Fas S., Schüttrumpf J., Henrichsen S. (2024). Comparative analysis of purity of human albumin preparations for clinical use. Anal. Chim. Acta.

[17] Grevys A., Nilsen J., Sand K.M.K., Daba M.B., Øynebråten I., Bern M., McAdam M.B., Foss S., Schlothauer T., Michaelsen T.E. (2018). A human endothelial cell-based recycling assay for screening of FcRn targeted molecules. Nat. Commun..

[18] Sand K.M., Bern M., Nilsen J., Noordzij H.T., Sandlie I., Andersen J.T. (2014). Unraveling the Interaction between FcRn and Albumin: Opportunities for Design of Albumin-Based Therapeutics. Front. Immunol..

[19] Farrugia A., Mori F. (2022). Therapeutic Solutions of Human Albumin—The Possible Effect of Process-Induced Molecular Alterations on Clinical Efficacy and Safety. J. Pharm. Sci..

[20] Steglich M., Lombide R., López I., Portela M., Fló M., Marín M., Alvarez B., Turell L. (2020). Expression, purification and initial characterization of human serum albumin domain I and its cysteine 34. PLoS ONE.

[21] Nagumo K., Tanaka M., Chuang V.T., Setoyama H., Watanabe H., Yamada N., Kubota K., Tanaka M., Matsushita K., Yoshida A. (2014). Cys34-cysteinylated human serum albumin is a sensitive plasma marker in oxidative stress-related chronic diseases. PLoS ONE.

[22] Bocedi A., Cattani G., Stella L., Massoud R., Ricci G. (2018). Thiol disulfide exchange reactions in human serum albumin: The apparent paradox of the redox transitions of Cys_34_. FEBS J..

[23] Lundahl M.L.E., Fogli S., Colavita P.E., Scanlan E.M. (2021). Aggregation of protein therapeutics enhances their immunogenicity: Causes and mitigation strategies. RSC Chem. Biol..

[24] Mikkat S., Dominik A., Stange J., Eggert M. (2020). Comparison of accompanying proteins in different therapeutic human serum albumin preparations. Biologicals.

[25] Marie A.L., Przybylski C., Gonnet F., Daniel R., Urbain R., Chevreux G., Jorieux S., Taverna M. (2013). Capillary zone electrophoresis and capillary electrophoresis-mass spectrometry for analyzing qualitative and quantitative variations in therapeutic albumin. Anal. Chim. Acta.

[26] Belousov A. (2019). Albumin and its Application. Mathews J. Case Rep..

[27] Oki S., Arakawa T., Shiraki K. (2016). The effects of N-acetyltryptophan and caprylic acid on protein aggregation. J. Biol. Macromol..

[28] Wu N., Liu T., Tian M., Liu C., Ma S., Cao H., Bian H., Wang L., Feng Y., Qi J. (2024). Albumin, an interesting and functionally diverse protein, varies from ‘native’ to ‘effective’. Mol. Med. Rep..

[29] Jalan R., Bernardi M. (2013). Effective albumin concentration and cirrhosis mortality: From concept to reality. J. Hepatol..

[30] Kalo E., Read S., Baig A., Marshall K., Ma W.S., Crowther H., Gofton C., Lynch K.D., Sood S., Holmes J. (2024). Efficacy of albumin use in decompensated cirrhosis and real-world adoption in Australia. JGH Open.

[31] Ma L., Jiang P., Wang Z., Liu Q., Xu J., Cheng L., Sun P., Du X., Li C. (2025). Protein composition analysis of human plasma-derived and recombinant human serum albumin preparations based on 4D label-free proteomics. PeerJ.

[32] Lichenstein H.S., Lyons D.E., Wurfel M.M., Johnson D.A., McGinley M.D., Leidli J.C., Trollinger D.B., Mayer J.P., Wright S.D., Zukowski M.M. (1994). Afamin is a new member of the albumin, alpha-fetoprotein, and vitamin D-binding protein gene family. J. Biol. Chem..

[33] Nomura K., Kawano K., Kawaguchi Y., Kawamura Y., Michibata J., Kuwata K., Sugiyama K., Kusumoto K., Futaki S. (2022). Hemopexin as a Potential Binding Partner of Arginine-Rich Cell-Penetrating Peptides in Serum. ACS Pharmacol. Transl. Sci..

[34] Wynendaele E., Ma G.J., Xu X., Cho N.J., De Spiegeleer B. (2021). Conformational stability as a quality attribute for the cell therapy raw material human serum albumin. RSC Adv..

