# Supplementary material for: Comparative Characterisation of Five Plasma-Derived Human Serum Albumin Preparations: Structural Integrity, Functional Properties, and Quality Attributes
Source: Pharmaceuticals (Basel). 2026 Sep 16;19(9):1470. doi: 10.3390/ph19091470 (PMC13610378; doi:10.3390/ph19091470)

Supplementary Materials

Table S1. Relative abundance of albumin proteins in HSA samples.

Product	Batch number	Albumin abundance (%)
VIALEBEX® 20%	23L04681	98.53
VIALEBEX® 20%	24L11734	98.28
ALBUNORM® 20%	P241A6862	98.91
ALBUNORM® 20%	P410B6865	98.69
ALBUTEIN® 25%	B02G049073	99.00
ALBUTEIN® 25%	B08G111353	98.73
FLEXBUMIN® 25%	LBO70524	99.53
FLEXBUMIN® 25%	LBO70698	99.02
ALBUREX® 20%	P100675741	98.29
ALBUREX® 20%	P100696807	98.96

Table S2. Analysed product batches.

Company	Product	Batch number	Concentration	Expiration date
LFB	VIALEBEX® 20%	23L04681	200 g/L	05/2026
LFB	VIALEBEX® 20%	24L11734	200 g/L	09/2027
Grifols	ALBUTEIN® 25%	B02G049073	250 g/L	06/2025
Grifols	ALBUTEIN® 25%	B08G111353	250 g/L	05/2025
Octapharma	ALBUNORM® 20%	P241A6862	200 g/L	09/2025
Octapharma	ALBUNORM® 20%	P410B6865	200 g/L	02/2027
Shire Baxalta	FLEXBUMIN® 25%	LBO70524	250 g/L	11/2024
Shire Baxalta	FLEXBUMIN® 25%	LBO70698	250 g/L	12/2024
CSL Behring	ALBUREX® 20%	P100675741	200 g/L	09/2026
CSL Behring	ALBUREX® 20%	P100696807	200 g/L	03/2027

Table S3. Excipient composition and sodium content of human serum albumin preparations.

Product	HSA conc.	Sodium (per 100 mL)	NaCl	Sodium caprylate / caprylic acid	N-acetyl-tryptophan(ate)	Other
VIALEBEX®	20%	280 mg	✓ (not quantified)	✓ (not quantified)	Absent	Water for injections
ALBUNORM®	20%	331–368 mg	5.7 g/L	2.3 g/L (as caprylic acid)	3.9 g/L (N-acetyl-DL-tryptophan)	Water for injections
ALBUREX®	20%	320 mg	✓ (not quantified)	✓ (not quantified)	✓ (not quantified)	Water for injections
FLEXBUMIN®	25%	~299–368 mg	4.3 g/L	2.7 g/L	4.3 g/L	Water for injections

ALBUTEIN®	25%	333.5 mg	*qs* to 0.145 mmol Na^+/mL	0.004 mmol/mL (~0.66 g/L)	0.004 mmol/mL (~1.07 g/L)	<2 mmol/L K^+; water for injections

conc: concentration; HSA: human serum albumin.

Table S4. Five VIALEBEX® batch analysis (%).

		VIALEBEX®
Analytical methods		
IEX (ion-exchange chromatography)	HSA -DA	5.9 ± 0.8
	Native HSA	39.1 ± 0.5
	HSA + Cys	26.9 ± 1.0
	HSA-DA-SO_2H	5.0 ± 0.1
	HSA + Cys-Gly	2.5 ± 0.1
	HSA-SO_2H	10.6 ± 0.5
SEC (size-exclusion chromatography)	Monomers	88.9 ± 1.11
	Dimers	2.2 ± 0.46
	Polymers	9 ± 0.83

Analysis performed with 5 different batches showing VIALEBEX® had low batch-to-batch variability; values represent mean ± SD. Cys: cysteinylated; Cys-Gly: cysteinylated and glycinylated; DA: truncated form; DA-SO_2H: truncated and oxidised; HSA: human serum albumin; Hex: glycated; SD: standard deviation; SO_2H: oxidised.

Figure S1. Surface plasmon resonance sensorgrams and residuals of albumin-FcRn interaction at pH 6.0

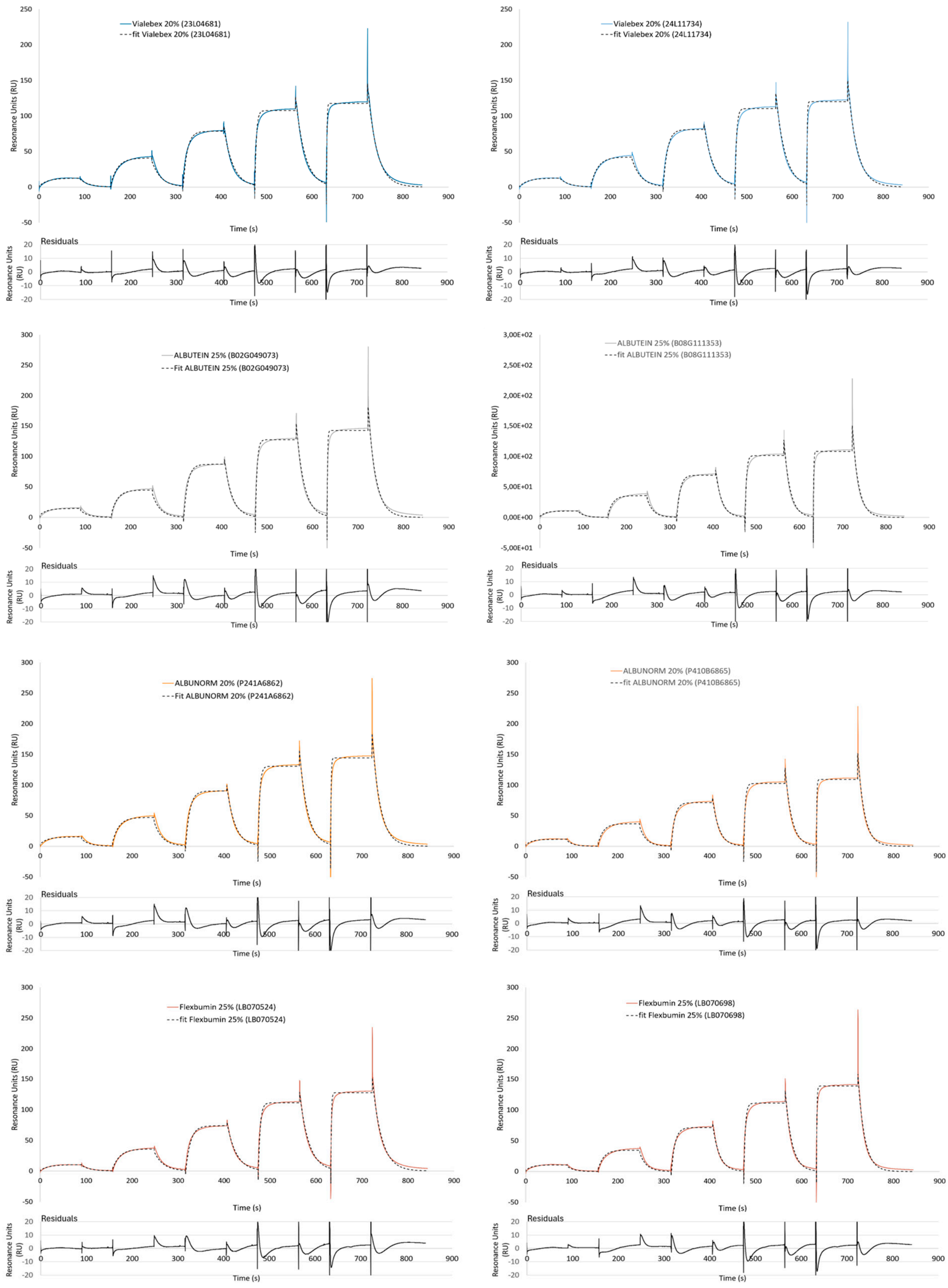
Vialebex 20% (23L04681)
fit Vialebex 20% (23L04681)
Vialebex 20% (24L11734)
fit Vialebex 20% (24L11734)
ALBUTEIN 25% (B02G049073)
Fit ALBUTEIN 25% (B02G049073)
ALBUTEIN 25% (B08G111353)
fit ALBUTEIN 25% (B08G111353)
ALBUNORM 20% (P241A6862)
Fit ALBUNORM 20% (P241A6862)
ALBUNORM 20% (P410B6865)
fit ALBUNORM 20% (P410B6865)
Flexbumin 25% (LB070524)
fit Flexbumin 25% (LB070524)
Flexbumin 25% (LB070698)
fit Flexbumin 25% (LB070698)
Resonance Units (RU)
Time (s)
Residuals

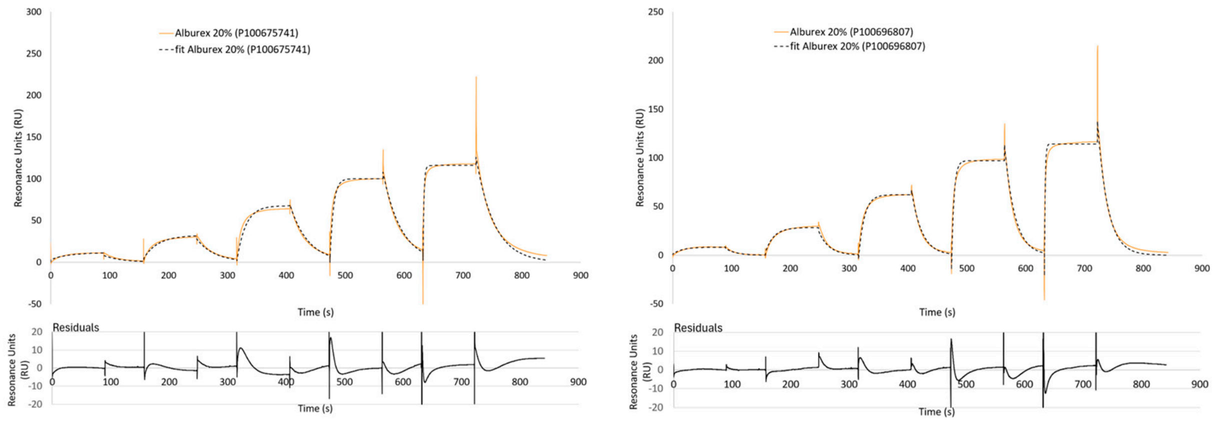

Figure S2. MS spectra of native, cysteinylated and oxidised albumin isoforms of VIALEBEX®. Inserts represent deconvoluted mass spectra.

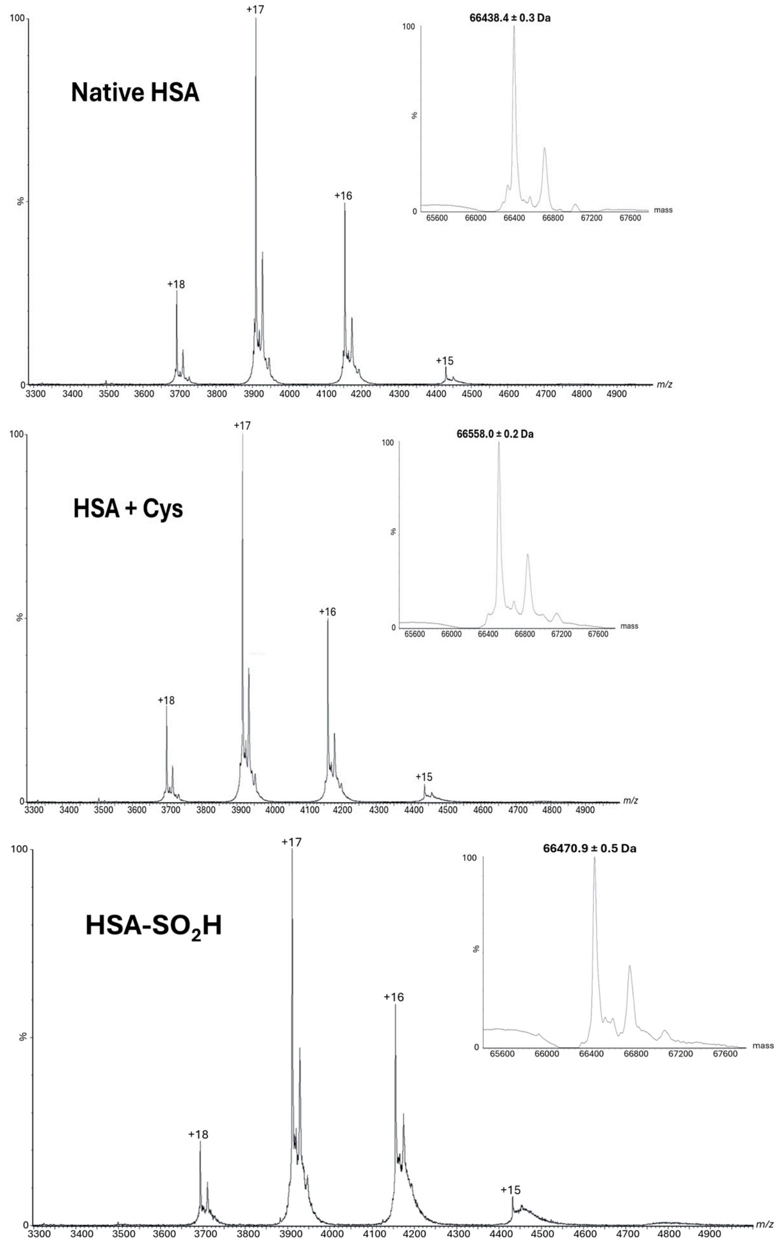

Supplement: Supplementary file 1 [file pharmaceuticals-19-01470-s001.zip › pharmaceuticals-4417229-supplementary.pdf]
